# Investigating the correlation between ultrasonic pulse velocity and compressive strength in polyurethane foam concrete

**DOI:** 10.1038/s41598-025-07666-3

**Published:** 2025-07-05

**Authors:** R. Roobankumar, M. SenthilPandian

**Affiliations:** School of Civil Engineering, Vellore Institute of Technology, Chennai, 600127 Tamil Nadu India

**Keywords:** Polyurethane foam waste, Compressive strength, Ultrasonic pulse velocity, Correlations, Concrete, Civil engineering, Mechanical properties

## Abstract

Using waste polyurethane foam as a partial replacement for natural coarse aggregates in concrete provides an eco-friendly solution by reducing waste and conserving natural resources. However, the strength behavior of polyurethane foam concrete differs from conventional concrete. To ensure effective design and quality control in the field, the viability of non-destructive testing methods for finding out the in situ mechanical properties of polyurethane foam concrete must be evaluated. This study establishes a correlation between compressive strength and ultrasonic pulse velocity (UPV) test to predict the compressive strength of polyurethane foam concrete using UPV test results. An experimental study was conducted on concrete specimens with varying percentages of polyurethane foam replacing natural coarse aggregate, ranging from 10 to 60% in 10% increments. The control concrete mix was 100% natural coarse aggregate without polyurethane foam. The properties of the specimens were evaluated after curing for 7, 14, and 28 days. It also examines polyurethane foam concrete workability, density, and microstructural properties. The findings show that the UPV and compressive strength of polyurethane foam concrete were lower than those of the control mix concrete for all replacement levels and curing ages. The empirical relationships between compressive strength and UPV were found to be exponential, with high correlation values ranging from 0.9012 to 0.9998. The predicted values and the experimentally measured results were compared in order to confirm the accuracy of the empirical equations for compressive strength prediction.

## Introduction

The construction sector contributes significantly to the excessive extraction of natural coarse aggregates from quarries to satisfy the ever-increasing demand for shelter and infrastructure development. This overexploitation of natural resources disrupts ecosystems and depletes finite reserves, resulting in long-term environmental consequences. Concrete, primarily composed of cement, water, fine and coarse aggregates, and chemical admixtures, is one of the most crucial components of modern construction. Its widespread use across construction projects highlights the need for sustainable construction practices and innovative alternatives to reduce the environmental impact^1^. Studies have examined the potential of various waste materials, including construction debris, agricultural residues, natural waste, quarry dust, recycled plastics, and hazardous waste, as replacements for aggregates, sand, cement, or their combinations in concrete^2–8^. This effort seeks to support sustainable development, enhance recycling practices, and contribute to environmental protection. Every year, millions of polyurethane foam waste are mainly generated by the construction, automotive, and refrigeration industries^9,10^. According to the most recent Statista report, the global polyurethane market volume was approximately in 2023 see about 26.22 million metric tons, and by 2030, that number is anticipated to rise to 31.27 million metric tons^11^. The building and construction sector consumes approximately 25% of polyurethane foam, followed by the automobile industry at 20%, refrigeration at 25%, and textiles along with other sectors accounting for the remaining 30%. Annually, polyurethane foam waste makes up about 30% of the market volume, with 22% disposed of in landfills, 33% recycled, and 45% incinerated^11,12^. Waste can be generated when polyurethane foam insulation materials are removed or replaced during construction, renovation, or demolition activities in buildings^13^. Waste may still result from contamination or unsuitability for recycling throughout the reprocessing or recycling phases, even during recycling initiatives^14–17^. Replacing natural coarse aggregates in concrete mixes with solid waste polyurethane foam is an eco-friendly and sustainable approach that promotes green construction methods. Using waste polyurethane foam as a partial replacement for coarse aggregate in concrete is essential for several reasons, including reducing waste from landfills, conserving resources by turning waste polyurethane foam into valuable building materials, lowering demand for conventional materials, which protects natural resources and lowers the environmental implications for processing and extraction, and lowering costs because waste materials are readily available and inexpensive^18–21^. Decreased transportation and disposal costs may also lead to significant cost reductions in waste management. The use of polyurethane foam waste as coarse aggregate in concrete is practical due to its wide availability as a byproduct from various industries, requiring minimal processing before use. Its lightweight nature makes it suitable for producing lightweight concrete. Numerous researchers studied the mechanical behavior, durability, and microstructure analysis of polyurethane foam as a substitute for natural coarse aggregate in concrete^22–25^. Their findings highlighted encouraging partial and complete replacement results, demonstrating its suitability for lightweight concrete structures. According to American Concrete Institute ACI 213R^26^lightweight concrete must achieve a minimum compressive strength of 17 MPa, while European Standard code EN 206 − 1^27^ defines structural lightweight concrete as having a maximum density of 2000 kg/m³.

Compressive strength is a key determinant of the performance of cementitious materials. It plays a fundamental role in determining the materials ability to withstand applied loads without failure. Additionally, several mechanical and durability characteristics, including elastic modulus, impermeability, and resilience to adverse weather conditions, are closely linked to compressive strength^28^. Compressive strength test data is frequently used to infer or estimate these characteristics, highlighting the significance of this information in evaluating the long-term dependability and functioning of cementitious materials^29^. The mechanical properties of concrete can be assessed through various methods, broadly categorized into destructive and non-destructive techniques. Destructive techniques require expensive, time-consuming, and highly specialized equipment. On the other hand, non-destructive methods offer a simple and affordable substitute for evaluating material characteristics^30^.

The Ultrasonic Pulse Velocity (UPV) method is a widely used non-destructive testing approach for assessing the structural integrity and mechanical properties of various materials^31^. It is particularly valued for its ability to provide quick, precise, and non-invasive assessments of materials such as concrete, mortar, bricks, masonry, backfill, cement paste, rock, and other similar materials^32–36^. The UPV approach is appropriate for both in-situ assessments and laboratory testing due to its adaptability. The UPV method measures the speed at which ultrasonic pulses pass through materials, mainly concrete, to evaluate their quality and characteristics. The method is based on the idea that a materials density, elasticity, cavities, cracks, or other internal flaws affect how quickly sound (ultrasonic pulses) travels through it. Non-destructive UPV methods determine material properties by establishing a correlation with specific, determinate experimental parameters.

Several studies have been conducted on the use of the UPV approach to assess the quality of concrete. Del Rio et al.^37^ reported an exponential relationship between concrete compressive strength and UPV using ultrasonic testing, resulting in a strength prediction model with a R² value of 0.92. Nash et al.^38^ investigated creating a consistent relationship between concrete compressive strength and non-destructive testing, revealing a better correlation between concrete compressive strength and UPV. Mohammed and Mahmood^39^ investigated the influence of maximum aggregate size on the UPV of concrete made with brick aggregates, finding a significant correlation between UPV, compressive strength, and the authors stated a strength prediction model with a correlation coefficient (R^2^) of 0.542 to 0.722. In their experimental work, Rao et al.^35^ investigated the UPV of roller-compacted concrete pavement that contained M-sand and fly ash. According to the findings, the UPV values of concrete mixes that included fly ash instead of cement increased with age. The reported UPV ranged from 2.8 to 4.42 km/s. Additionally, the authors developed a strength prediction model using UPV analysis, achieving a correlation coefficient (R²) of 0.895. A study by Wang et al.^40^ examined the application of UPV in waste glass concrete that self-consolidates. They discovered that as concrete aged, its strength increased, but as the water-to-binder ratio increased, it reduced. Al-Numan et al.^41^ utilized UPV measurements to create a formula for predicting the compressive strength of single-grade concrete. Moreover, Demirboga et al.^42^ explored the correletion between compressive strength and UPV in high-volume mineral-admixtured concrete. A determination coefficient (R^2^) of 0.96 suggests a very excellent exponential connection between UPV and compressive strength when all values are combined. Shariq et al.^43^ studied the UPV of concrete containing ground granulated blast furnace slag and discovered that the relationships between UPV and compressive strength could effectively predict the compressive strength of ground granulated blast furnace slag concrete at any replacement level. The results indicate a strong correlation between the UPV and compressive strength of conventional concrete mixes. Among ground granulated blast furnace slag concrete mixes, those containing 40% ground granulated blast furnace slag exhibited a better correlation between compressive strength and UPV compared to mixes with 20% or 60% ground granulated blast furnace slag.

Recently, several studies have analyzed the correlation between UPV and the compressive strength of lightweight aggregate concrete. Fatahi et al.^44^ investigated the prediction of lightweight aggregate concrete compressive strength. The strength varied between 7.6 and 16.47 MPa, with an UPV of 2–3.2 km/s. The authors used UPV analysis to develop a strength prediction model with a correlation coefficient (R^2^) of 0.81. Nachiar et al.^45^ investigated the correlation between fly ash cenosphere concrete compressive strength and UPV. The strength ranged from 24.1 to 34.13 MPa, with an UPV of 3.65–4.89 km/s. Using UPV analysis, the authors stated a strength prediction model with a correlation coefficient (R^2^) of 0.91 to 0.99. Shafigh et al.^46^ studied prediction of lightweight aggregate concrete using limsstone powder and oil palm shell. This resulted in a compressive strength ranging from 5.8 to 41.5 MPa and an UPV of 3.71 to 3.89 km/s. Based on their UPV results, the authors proposed a strength prediction model with an R² value of 0.94. Semiha et al.^47^ investigated lightweight concrete incorporating waste PET lightweight aggregate as a replacement for conventional aggregate. They reported a 28-day compressive strength ranging from 9.5 to 25.3 MPa and an UPV between 2.44 and 3.6 km/s. Additionally, the study presented a strength prediction model with an R² value of 0.93. Nikbin et al.^48^ investigated lightweight concrete by combining expanded clay aggregate with red soil. The authors proposed a strength prediction model with R^2^ = 0.84, and they reported a strength in the range of 18.83–28.83 MPa and an UPV of 3.7–4.3 km/s. Y. Zhang and F. Aslani^49^ investigated compressive strength prediction models for lightweight aggregate concretes utilising UPV. The authors stated a lightweight aggregate concrete strength prediction model with an R^2^ of 0.75. Majhi et al.^50^ investigated lightweight aggregate concrete using sintered fly ash. They reported a 28-day compressive strength ranging from 11.3 to 35.3 MPa and an UPV between 3.2 and 5 km/s. Additionally, the study presented a strength prediction model with an R² value of 0.96. Kim et al.^51^ discovered a correlation between UPV and compressive strength in lightweight aggregate concretes, resulting in a strength prediction model with a R² value of 0.65 to 0.97. Recently, M. Belmokaddem et al.^52^ studied lightweight concrete by replacing traditional aggregate with waste plastic. They reported a 28-day compressive strength ranging from 4 to 26.9 MPa and an UPV between 2.6 and 4.8 km/s. The study also presented a strength prediction model with a strong correlation, showing an R² value between 0.89 and 0.98. A comparison with actual test results validates the accuracy of these empirically developed equations for compressive strength.

The development of using waste polyurethane foam as coarse aggregate in concrete mixes. Previous research has mostly used a polyurethane foam as a fine aggregate in concrete to improve mechanical performance. There are limited studies on using waste polyurethane foam as coarse aggregate in concrete. The present study utilizes high-density rigid polyurethane foam as a coarse aggregate, leveraging its high closed-cell structure to reduce porosity and enhance compressive strength. The primary objective is to develop polyurethane foam waste as a coarse aggregate in concrete, achieving higher compressive strength than previous studies. This advancement aims to increase the utilization of waste polyurethane foam in the construction sector, promoting sustainability and efficient waste management. According to earlier research, the UPV test is frequently used to describe the characteristics of concrete. Polyurethane foam concrete is an environmentally friendly material with much promise for application in building construction. Numerous physical, mechanical, microstructural, and durability features of polyurethane foam concrete have been investigated in earlier research. However, there is a lack of studies that focus on predicting the compressive strength of polyurethane foam concrete mixes before construction. To address this gap, this study focused on formulating predictive equations derived from non-destructive UPV testing through experimental analysis. An experimental study has been conducted on concrete specimens that were examined with different percentages of polyurethane foam replacing natural coarse aggregate at 10%, 20%, 30%, 40%, 50%, and 60%. Their properties were evaluated after curing for 7, 14, and 28 days. Experimental tests were conducted to assess fresh concrete properties using the slump cone test and hardened concrete characteristics through compressive strength, density, and UPV tests. Additionally, scanning electron microscopy (SEM) with Energy Dispersive X-ray Analysis (EDAX) analysis was performed to examine the morphology, voids, aggregate-matrix interface, and chemical composition. The test results were analyzed, and empirical equations were proposed to predict the compressive strength of polyurethane foam concrete using UPV data. These equations were further validated against experimental results to ensure their accuracy.

## Materials and testing methodology

### Materials characterization and mix proportions

The characteristics of the concrete mix, including cement, water, fine and coarse aggregate, polyurethane foam aggregate, and polycarboxylate-based superplasticizer, complied with the requirements outlined in the relevant Indian Standard (IS) guidelines. IS 12,269 was followed by ordinary Portland cement with grade 53^53^. Table 1 lists the cement initial and final setting times, finess, and standard consistency, which were determined in accordance with IS 4031 (Parts 4 and 5)^54,55^. The coarse aggregate consisted of crushed granite blue metal sourced from local quarries, with particle sizes ranging from 4.75 to 20 mm, while the fine aggregate was manufactured sand (M sand), with a maximum particle size of 4.75 mm. Polyurethane foam waste was collected from Sri Prasanna Energy System and Services, a locally available manufacturing company in Chennai, India.

**Table 1:** Physical properties of cement.

Initial setting time (minutes)	Final setting time (minutes)	Specific gravity	Normal consistency (%)	Fineness (%)
65	350	3.15	30	1.66

**Fig. 1:**
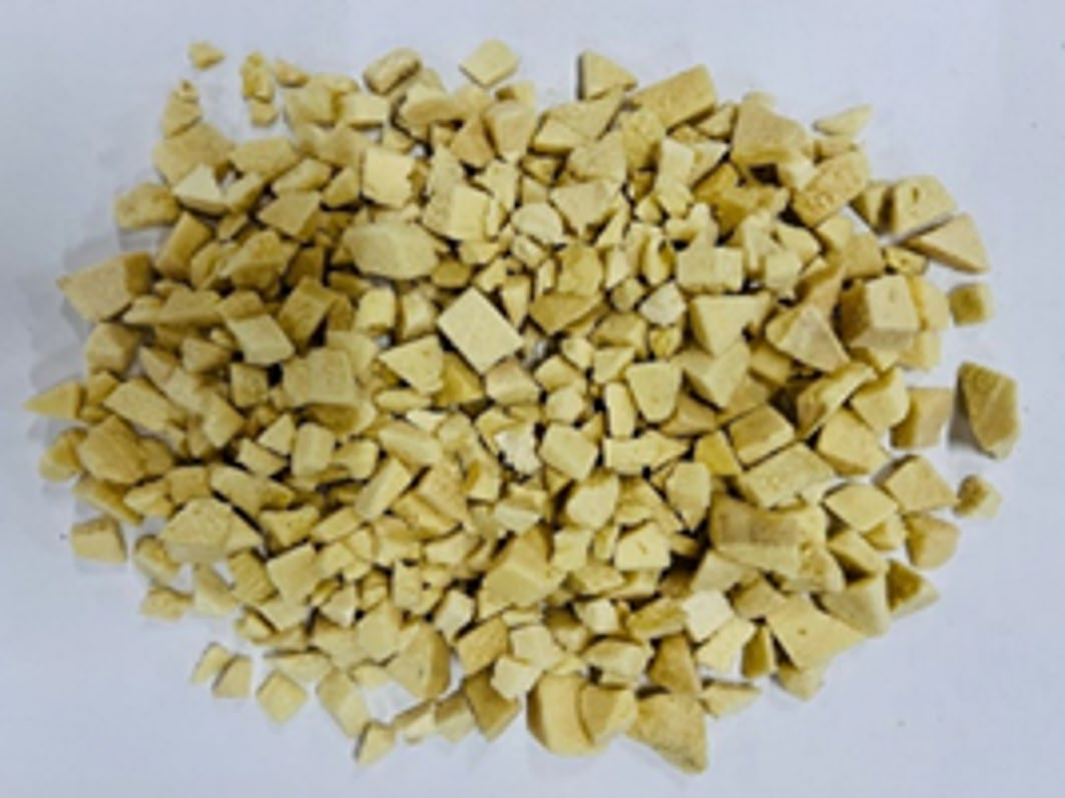
Polyurethane foam as coarse particles.

**Fig. 2:**
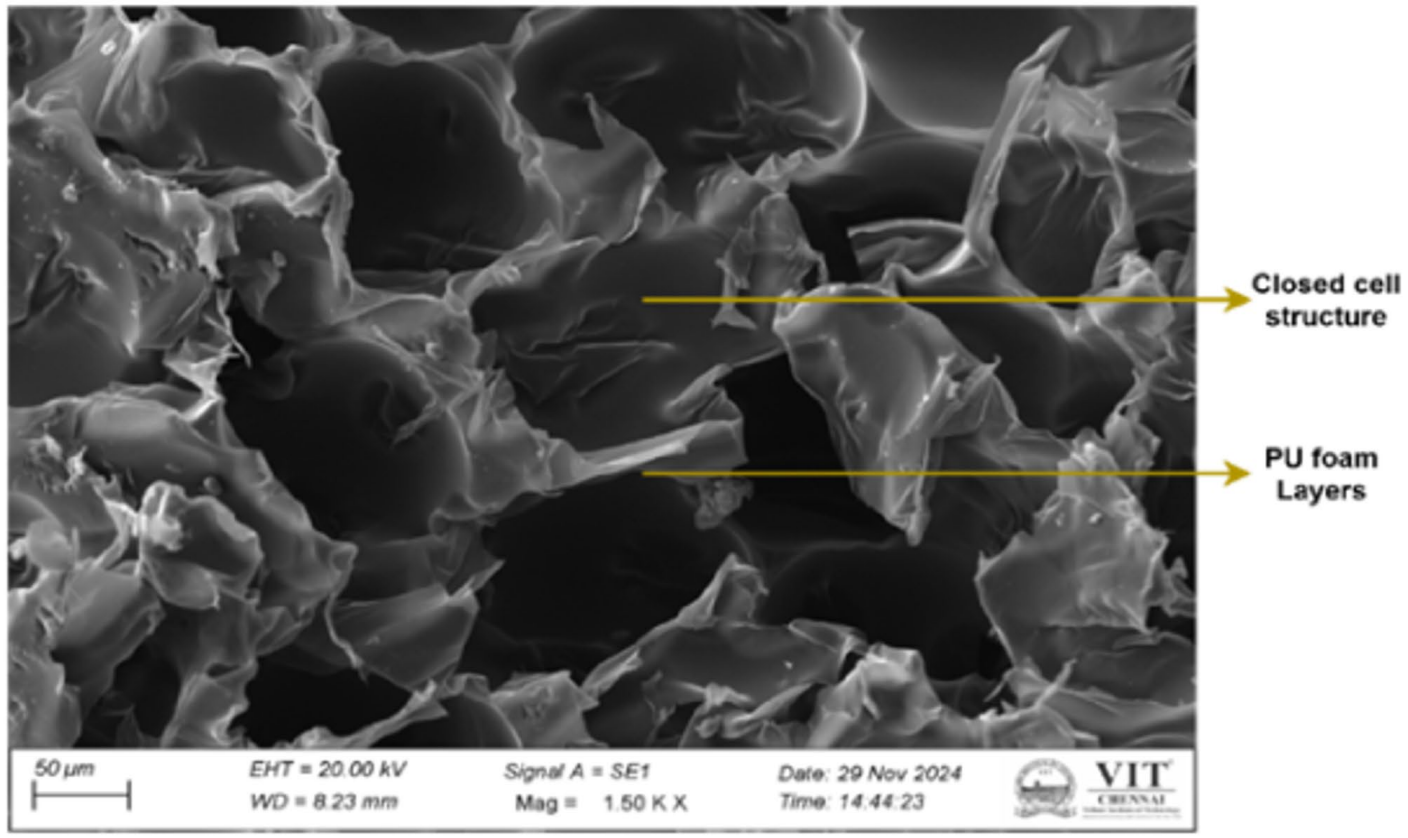
SEM analysis of the polyurethane foam.

**Fig. 3:**
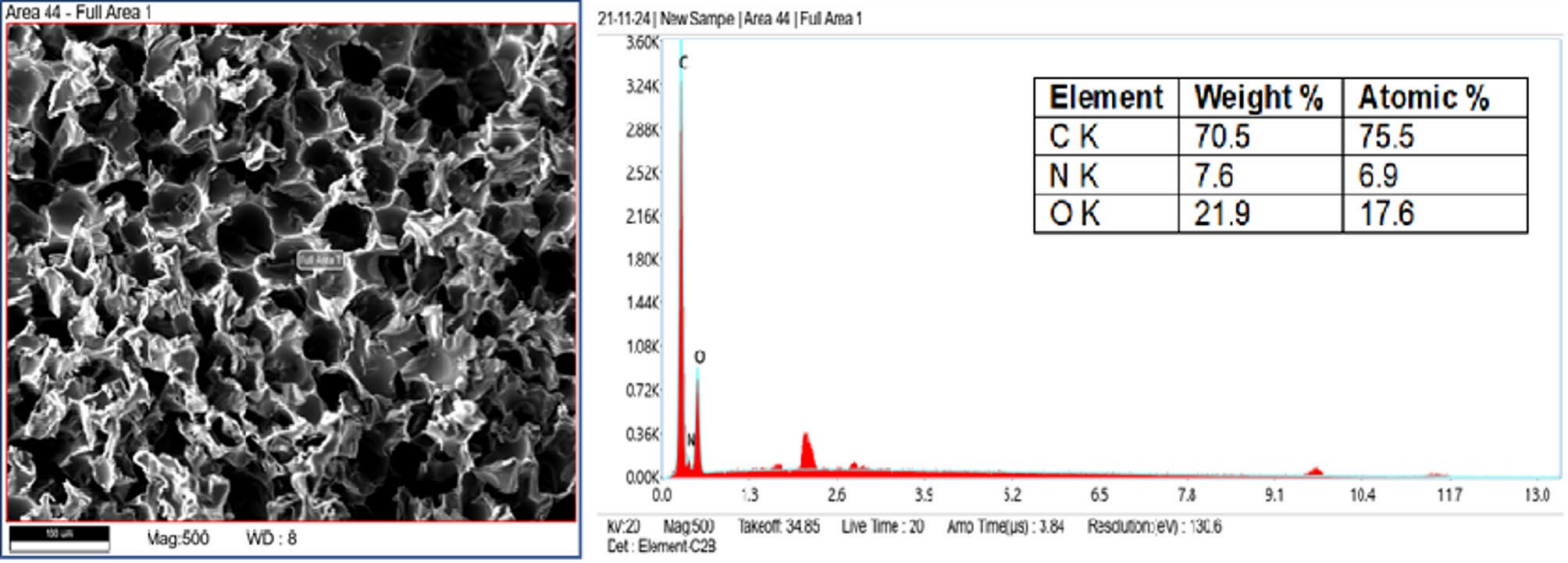
SEM-EDX analysis of the polyurethane foam.

A producer provided waste panels and scraps, as shown in the Fig. 1, which were then crushed into coarse particles in a polyurethane foam recycling lab. Waste polyurethane foam, ranging in size from 4.75 to 20 mm, was utilized in concrete with different volume substitutions for coarse aggregate. Potable water from the locale was utilized for curing and mixing. A chemical admixture of superplasticizers based on polycarboxylate was added to the concrete mixture to increase workability. The material has a dry content of 21% and a density of 1050 kg/m³. In compliance with IS 2386 (Part 3)^56^ tests were performed to determine the specific gravity, density, and water absorption of coarse aggregate, fine aggregate, and polyurethane foam aggregate. However, to ensure the polyurethane foam aggregate sample was submerged and not floating, it was placed inside the container filled with water. The polyurethane foam was held underwater by a wire mesh and cloth, which stopped it from floating because it was porous and compressible. Table 2 displays the physical properties of waste polyurethane foam, natural coarse, and manufacturing sand. A SEM image of polyurethane foam is displayed in Fig. 2. In contrast to natural coarse aggregate, polyurethane foam is highly porous with a rough surface. SEM observations also show that rigid polyurethane foam has a closed-cell structure. Figure 3 represents the SEM with EDAX of polyurethane foam, revealing that carbon is the major component.

**Table 2:** The physical properties of waste polyurethane foam, natural coarse, and manufacturing sand.

Property	Density (kg/m^3^)	Water absorption 24 h (%)	Specific gravity
Polyurethane foam coarse aggregate	200	13.5	0.32
Natural coarse aggregate	1705	1.2	2.75
M sand	1745	1.04	2.65

A machine concrete mixer was used to prepare six different concrete mix proportions; the first mix served as the control mix, and waste polyurethane foam was added to the other five mixes. Polyurethane foam was used to replace natural coarse aggregate in varying volume proportions. The natural coarse aggregate was replaced at percentages of 10% (M1), 20% (M2), 30% (M3), 40% (M4), 50% (M5), and 60% (M6). The mix proportions were designed following the guidelines of IS 10,262^57^, as detailed in Table 3.

**Table 3:** Mix proportions.

Mix	Cement (kg/m^3^)	Water (kg/m^3^)	M sand (kg/m^3^)	Coarse aggregate (kg/m^3^)	Polyurethane foam aggregate (kg/m^3^)	Polyurethane foam replacement level by coarse aggregate volume (%)	Superplasticizer (kg/m^3^)	W/c ratio
CM	340	152	842	1145	-	-	5.1	0.45
M1	340	152	842	1030.5	13.43	10	5.1	0.45
M2	340	152	842	916	26.86	20	5.1	0.45
M3	340	152	842	801.5	40.29	30	5.1	0.45
M4	340	152	842	687	53.72	40	5.1	0.45
M5	340	152	842	572.5	67.15	50	5.1	0.45
M6	340	152	842	458	80.58	60	5.1	0.45

## Methods of testing

The consistency of fresh concrete was assessed using the slump test in accordance with IS 7320. In order to improve flowability and avoid floating during mixing, pre-wetted polyurethane foam waste was utilized for coarse aggregate in concrete^58,59^. By pre-saturating the polyurethane foam aggregates before mixing, we ensured that their absorption did not affect the free water content in the concrete mix. In accordance with IS 516^60^, a concrete cube of 100 mm by 100 mm by 100 mm was made for compressive strength. The specimens were placed in the middle of the compressive testing device after curing for 7, 14, and 28 days. Until the specimen failed, a load was applied at a rate of 13.7 N/mm²/min, and the highest load was noted. The greatest force was then divided by the specimens cross-sectional area to determine its compressive strength. The UPV test measures the speed at which ultrasonic pulses pass through concrete to assess its strength, homogeneity, and quality without causing any damage. It aids in evaluating the overall integrity of the concrete and identifying interior defects, cracks, and voids. In accordance with IS 13,311 (Part 1)^61^ guidelines, the concrete quality was evaluated using the UPV method. A grease oil was placed after the surface had been carefully cleaned to ensure sufficient acoustic contact between the transducers and the concrete surface. The UPV was measured using a 54 kHz transducer with a precision of 0.1 µs, connected to a UPV testing apparatus (Proceq Pundit Lab). Prior to testing, the device was calibrated with a reference bar to eliminate any instrumental errors.

The transducers were positioned appropriately, and ultrasonic pulses were transmitted through the concrete element. The time taken for the pulses to travel between the transmitting and receiving transducers was recorded. The pulse velocity was calculated using the recorded travel time and path length to assess the concrete quality. Concrete microstructural characteristics are investigated via SEM image examination. It aids in analyzing hydration products, fractures, surface morphology, pore structure, and aggregate-cement matrix bonding^62,63^. The center portion of the small piece (less than 1 cm) of hardened concrete was taken for micro-level analysis.

## Results and discussions

### Workability

**Fig. 4:**
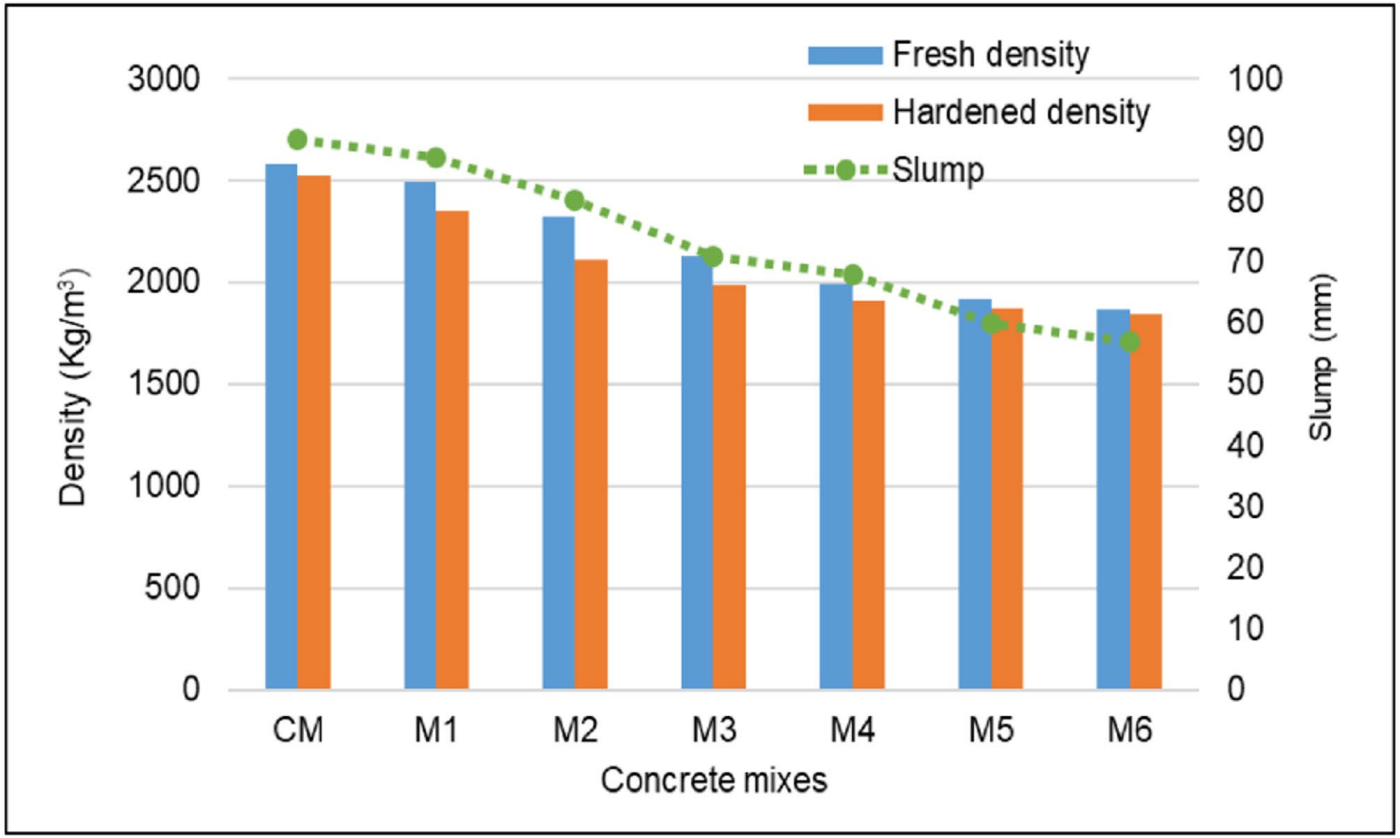
Density and slump of polyurethane foam concrete.

Workability measures how easily freshly mixed concrete can be placed, compacted, and finished without segregation or loss of homogeneity. The workability of fresh concrete was evaluated using the slump test, conducted in compliance with IS 7320 standards^58^. In addition to offering an early warning of possible variations in mix qualities, this test is crucial for ensuring the consistency and quality of concrete during construction. Slump values can be tracked to determine whether concrete is appropriate for a given application and ensure that project parameters are followed. This study evaluated the fresh properties of concrete mixtures that partially substituted waste polyurethane foam for natural coarse aggregate. The polyurethane foam replaced 10%, 20%, 30%, 40%, 50%, and 60% of the natural coarse aggregate by volume. In order to improve flowability and minimize floating while mixing, concrete was made using pre-wetted polyurethane foam waste as coarse aggregate. Figure 4 shows that the slump value of concrete decreases gradually as the polyurethane foam content rises, owing to its highly porous nature. From Fig. 4, the control mix concrete slump value was 90 mm. In comparison, the slump values for the mixes chosen as M1, M2, M3, M4, M5, and M6 were 87 mm, 80 mm, 71 mm, 68 mm, 60 mm, and 57 mm, respectively. Compared to the control mix concrete, these figures indicated reductions in workability of 3.4%, 11.2%, 21.2%, 24.5%, 33.4%, and 36.7%, respectively. Polyurethane foam particles were more porous than natural coarse aggregate, allowing the concrete mix to absorb the available water. The reduction in slump value with polyurethane foam in concrete is due to its high porosity, which absorbs more water, reducing free water in the mix. Its low density, irregular shape, and increased surface area disrupt cohesiveness and packing efficiency, further lowering workability.

### Density

Concrete density is important since it directly affects the materials strength, durability, and overall performance. It plays a critical part in determining the suitability of concrete for specific structural and non-structural applications^64,65^. Figure 4 illustrates that the slight decrease in the density of hardened concrete compared to fresh concrete occurred due to water evaporation, the retention of some air voids, and shrinkage from drying or thermal contraction. Additionally, the hydration process caused minor expansion, and further water loss from evaporation reduced the density. According to Fig. 4, the 28-day concrete containing polyurethane foam had a lower hardened density than the control mix. M6, which used 60% polyurethane foam as its coarse aggregate, has the lowest hardened density of all the mixes in the series. The control mix concrete had a hardened density of 2585 kg/m^3^. The mixes identified as M1, M2, M3, M4, M5, and M6 had hardened density values of 2354 kg/m^3^, 2110 kg/m^3^, 1987 kg/m^3^, 1895 kg/m^3^, 1870 kg/m^3^, and 1842 kg/m^3^, respectively. The hardened density reductions of 6.7%, 16.4%, 21.3%, 24.9%, 25.9%, and 27%, respectively, in comparison to the control mix concrete. As per ACI 213R, lightweight aggregate concrete density ranges from 1120 to 1920 kg/m^3^. According to EN 206 − 1^27^, structural lightweight concrete has a maximum density of 2000 kg/m^3^. According to the results of this study, concrete with polyurethane foam contents of 30%, 40%, 50%, and 60% met the requirements for structural lightweight concrete.

### Microstructure analysis

**Fig. 5:**
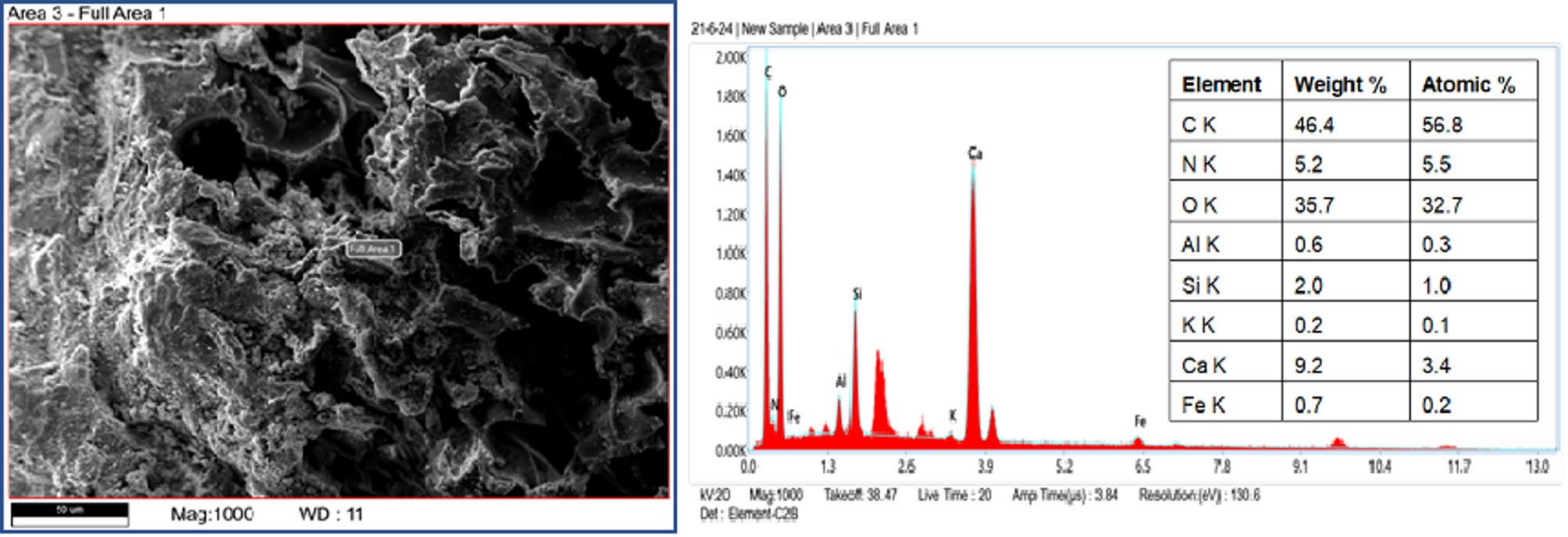
SEM-EDX analysis of the polyurethane foam concrete.

**Fig. 6:**
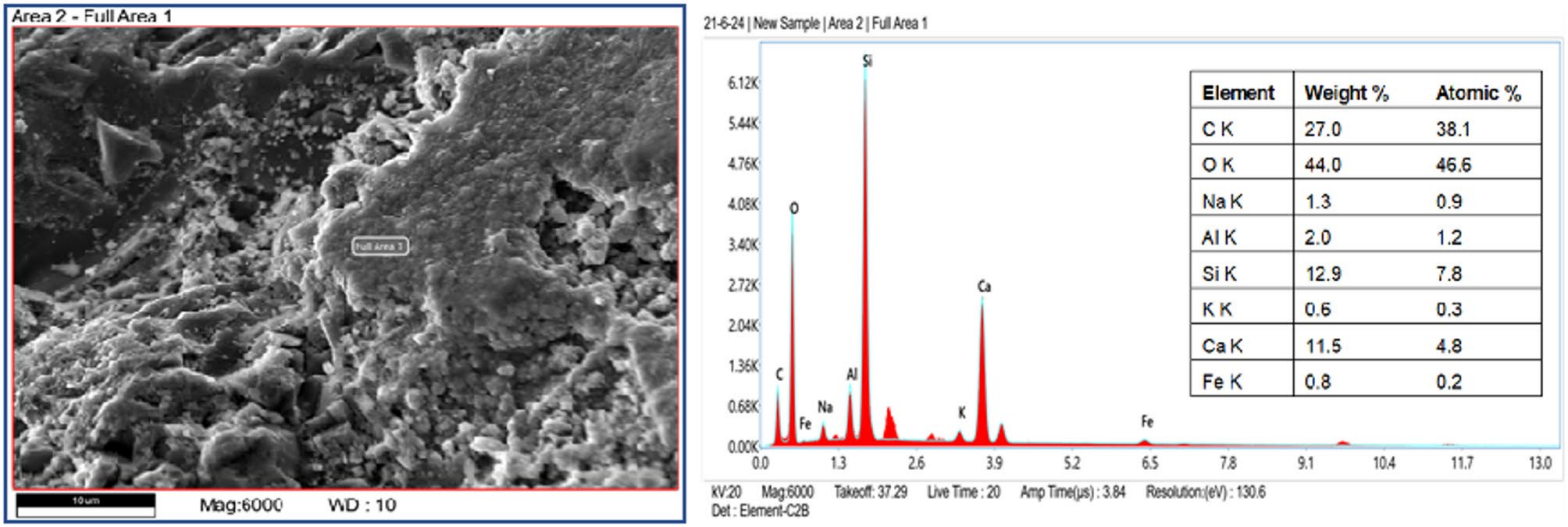
SEM-EDX analysis of the normal concrete.

SEM provides detailed images of surface morphology, pore structure, and the interfacial transition zone (ITZ), helping to understand the void distribution, crack patterns, and bonding between aggregates and the cementitious matrix^66,67^. EDAX complements this by identifying the chemical elements, verifying the distribution of additives, and detecting impurities^68,69^. After 28 days of curing, small pieces of polyurethane foam concrete were extracted from the center of the specimen and prepared for SEM testing. The elemental composition of polyurethane foam concrete is depicted in EDAX Fig. 5, which emphasizes the presence of organic polymeric foam and the dominance of carbon (C) and oxygen (O). Strength and durability are enhanced by calcium (Ca), silicon (Si), and aluminum (Al), which reflect the cementitious matrix. Iron (Fe) and potassium (K), two minor metals, indicate additions or contaminants. This hybrid composition combines lightweight, insulating properties from the foam with structural integrity from the cementitious materials, making it suitable for lightweight construction applications. Figure 6 EDAX graph illustrates the composition of standard concrete, highlighting the dominance of calcium, silicon, and oxygen, which reflect the cement and aggregate components. Minor elements such as potassium and sodium influence durability and may contribute to alkali-silica reactions. Adding polyurethane foam, an organic polymer, is the leading cause of the greater carbon content in polyurethane foam concrete compared to conventional concrete.

**Fig. 7:**
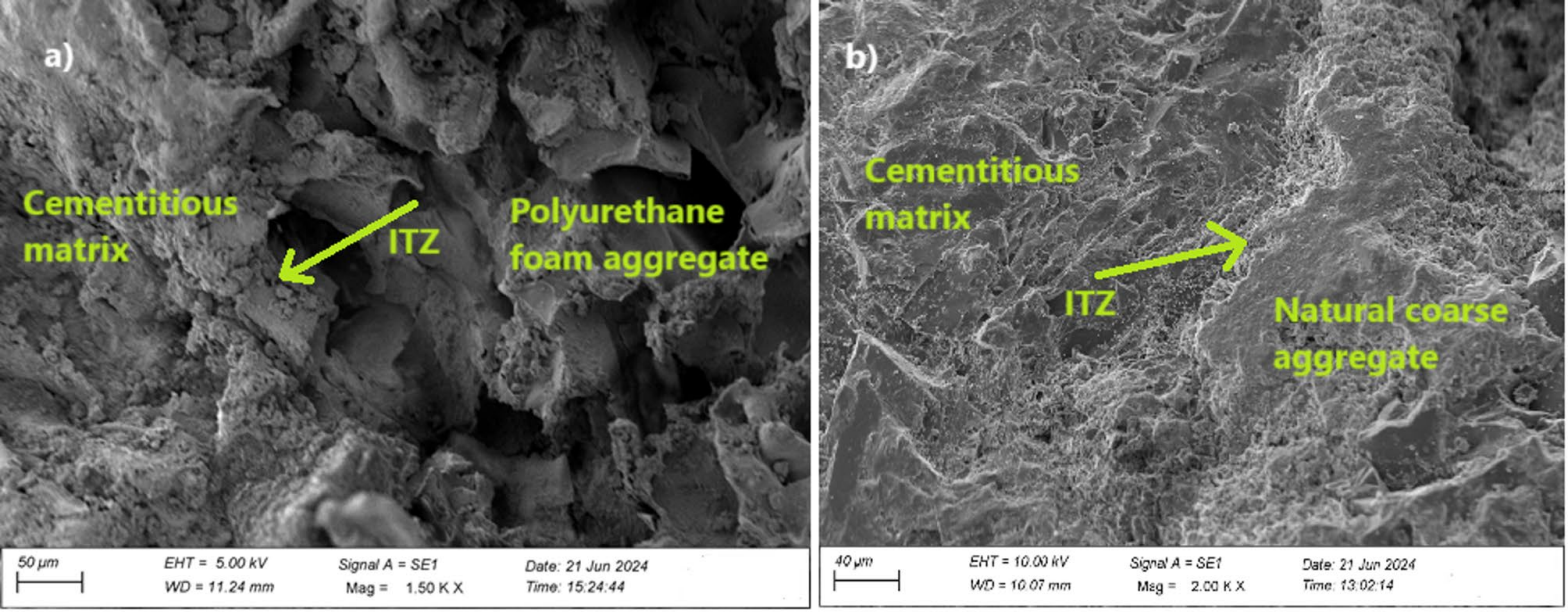
SEM image shows the ITZ behavior (a) polyurethane foam concrete and (b) conventional concrete.

In particular, lightweight concrete, which uses lightweight particles to lower density while keeping strength, depends heavily on the ITZ when establishing the overall characteristics of the material. Figure 7 represents the SEM images of polyurethane foam concrete, illustrating the ITZ behavior with improved bonding between the polyurethane foam aggregate and the cementitious matrix. This enhanced bonding was attributed to the polyurethane foam high porous and surface porosity. The high porous allowed the cement paste to penetrate the surface, strengthening the bond with the polyurethane foam. As reported by Mounanga et al.^22^ and Ben Fraj et al.^23^good adhesion between the cementitious matrix and the polyurethane foam aggregates is attributed to the penetration of cement paste into the surface porosity of the polyurethane foam.

### Compressive strength and UPV

#### Influence of polyurethane foam/replacement level

**Fig. 8:**
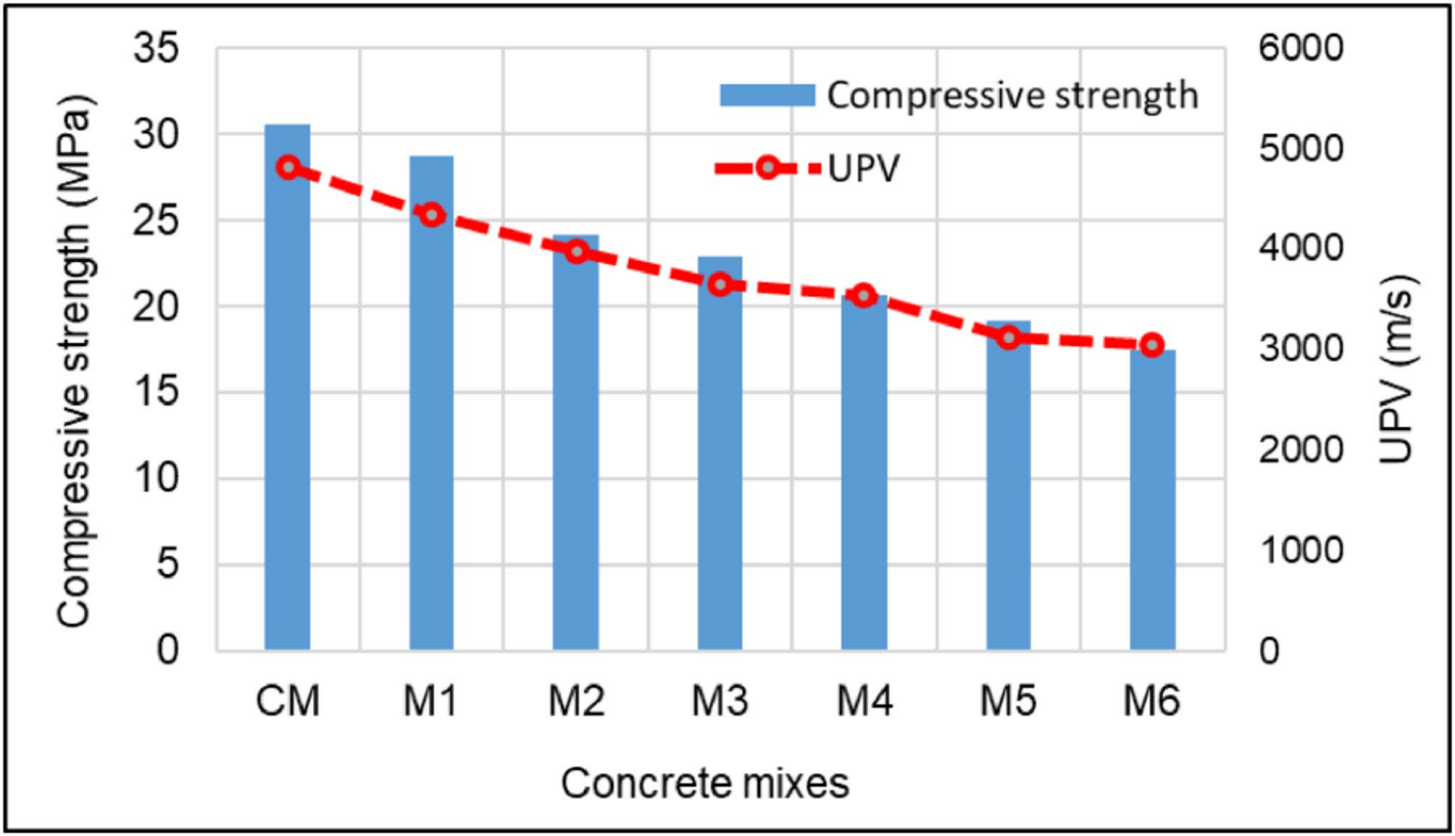
Compressive strength and UPV of concrete specimens with different levels of polyurethane foam replacement.

Figure 8 shows the variation of compressive strength and UPV for different concrete mixes. To make the study analysis easier, the UPV and compressive strength test results of hardened concrete at a 28-day curing age were utilized as reference data. The 28-day curing period resulted in higher compressive strength than 7 or 14 days. It allowed more time for the hydration process to progress, leading to better matrix maturity, densification, and strength development. Engineers frequently used test results from earlier studies to make well-informed decisions depending on the particular requirements of a project, even though a 28-day testing period was typically employed to assess the design strength of concrete. The compressive strength of the control mix (CM), which did not include polyurethane foam replacement, was the highest at 30.5 MPa. After substituting the coarse aggregate with polyurethane foam at different volume percentages, the 28-day compressive strength of the concrete mixtures was 28.7 MPa for 10% substitution (M1), 24.2 MPa for 20% substitution (M2), 22.9 MPa for 30% substitution (M3), 20.6 MPa for 40% substitution (M4), 19.1 MPa for 50% substitution (M5) and 17.4 MPa for 60% substitution (M6). The compressive strength decreased consistently as the polyurethane foam replacement increased progressively from M1 to M6. Mix M1 exhibited a slightly lower compressive strength than the control mix, while Mix M5 & M6 demonstrated the most significant reduction, with a compressive strength of less than 20 MPa. The compressive strength of concrete experienced a minor reduction when 10% and 20% of the coarse aggregate was replaced with polyurethane foam. The compressive strength was reduced by 24.9%, 32.46%, 37.38, and 42.95%, respectively, as the amount of polyurethane foam increased by 30, 40, 50, and 60%. As the proportion of polyurethane foam increased to 30%, 40%, 50%, and 60%, the concrete compressive strength decreased by 24.9%, 32.46%, 37.38%, and 42.95%, respectively. The decrease in concrete compressive strength was attributed to the lower density, higher porosity, inferior mechanical properties, and load-bearing capacity of polyurethane foam compared to natural coarse aggregates. As the polyurethane foam content increases, the matrix becomes weaker, and the volume of air voids increases, further reducing strength. However, compared to previous studies, the compressive strength of polyurethane foam lightweight concrete increased by 43% due to the use of high-density rigid polyurethane foam^22,23^. The penetration of cement paste into the surface porosity of the polyurethane foam aggregate enhanced bonding properties, leading to improved compressive strength. According to ACI 213R, lightweight concrete must have a minimum compressive strength of 17 MPa. In this study, all mix proportions that achieved a 28-day compressive strength exceeding 17 MPa met the criteria for structural lightweight concrete. Additionally, EN 206-1 defines structural lightweight concrete as having a maximum density of 2000 kg/m³. Based on the results, concrete mixes containing 30%, 40%, 50%, and 60% polyurethane foam satisfied both the density and strength requirements for structural lightweight concrete.

As per IS 13,311 (Part 1): 1992^61^, The UPV test measured the speed at which ultrasonic pulses passed through concrete to evaluate its quality. The quality of concrete was classified based on the pulse velocity: values above 4500 m/s indicated excellent quality, 3500–4500 m/s denoted good quality, 3000–3500 m/s suggested medium quality and velocities below 3000 m/s indicated doubtful or poor quality. As seen in Fig. 8, polyurethane foam concrete UPV values were continuously reduced compared to those of the control mix concrete. The UPV values of the 28-day cured polyurethane foam concrete samples range from 4341 to 3042 m/s, which is less than the UPV value of the control mix concrete, which is 4810 m/s. The UPV values of concrete containing 10%, 20%, 30%, 40%, 50%, and 60% polyurethane foam were decreased by 9.75%, 17.36%, 24.3%, 26.46%, 35.17%, and 36.75%, respectively. These lower UPV values in polyurethane foam concrete resulted from the lower density, voids, and weaker mechanical properties of polyurethane foam aggregates, which decreased the travel velocity of the waves. The UPV value exceeds 3000 m/s across all mix proportions, meeting the requirements of IS 1331^61^ for good and excellent quality concrete. High-density rigid polyurethane foam with a more closed-cell structure tends to result in higher UPV values due to its reduced air void content.

#### Influence of curing period

**Fig. 9:**
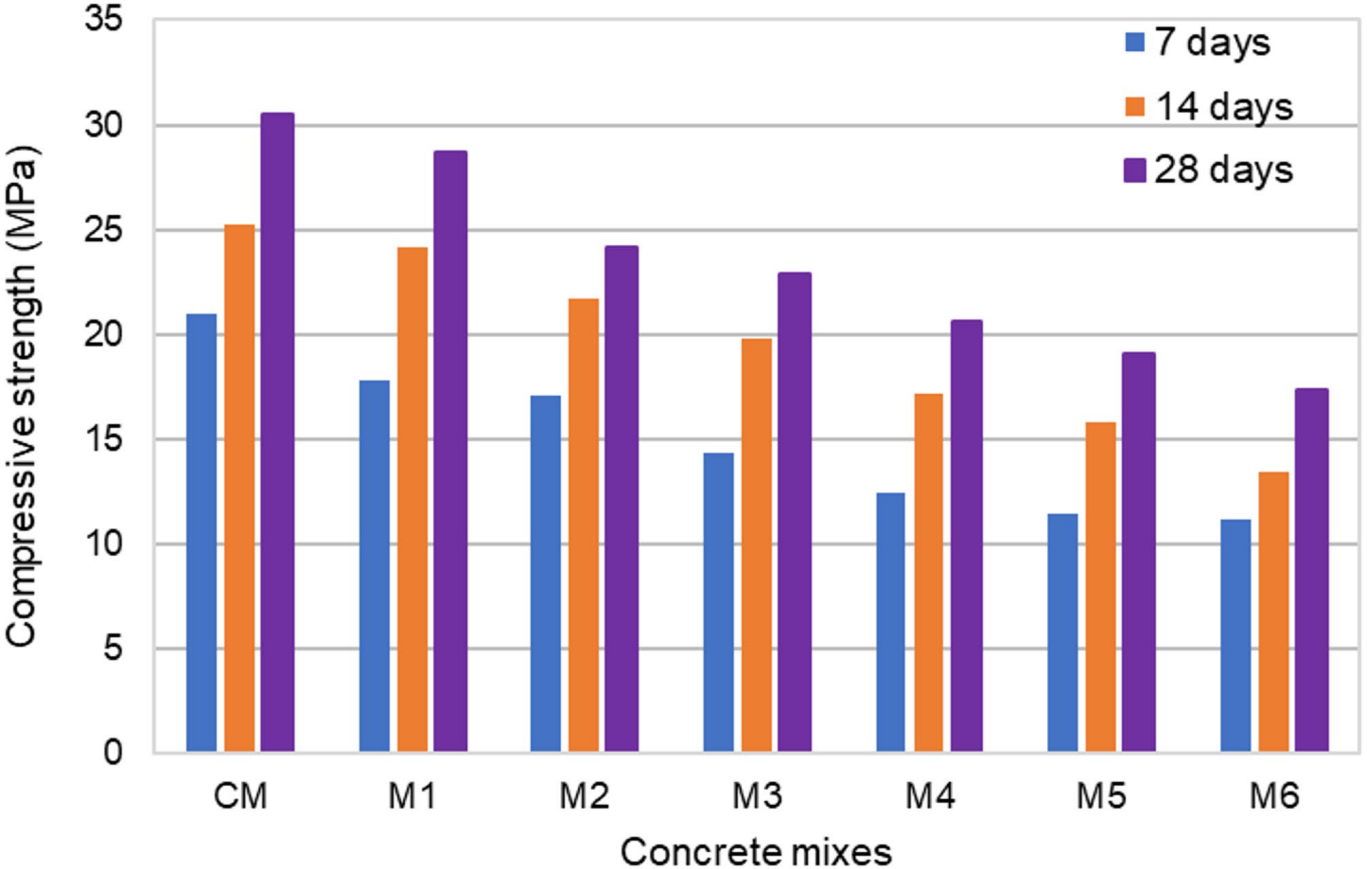
Compressive strength at various curing periods.

Figure 9 illustrates the results of polyurethane foam concrete compressive strength tests conducted at different curing periods. As anticipated, the compressive strength improved as curing periods increased. This improvement is explained by the hydration products that continue to form over time, strengthening the connection between the particles. As the curing period extends, the concrete undergoes further hydration, leading to a more densely packed microstructure. More extended curing periods contribute to a developed pore structure, making the concrete less permeable. This reduced permeability develops the concrete mechanical properties, making it more resistant to water infiltration and various ecological factors. Furthermore, the variation in composition among different concrete mixes influences the differences in strength development and the degree of development observed in the specimens. It was shown that between 7 and 28 days, the concrete compressive strength increased substantially. Furthermore, it was discovered that the compressive strength of polyurethane foam concrete was less than that of the control mix concrete for every replacement level. The compressive strength of polyurethane foam concrete decreased due to the high water content, higher porosity, and the replacement of dense natural coarse aggregates with high porous polyurethane foam.

**Fig. 10:**
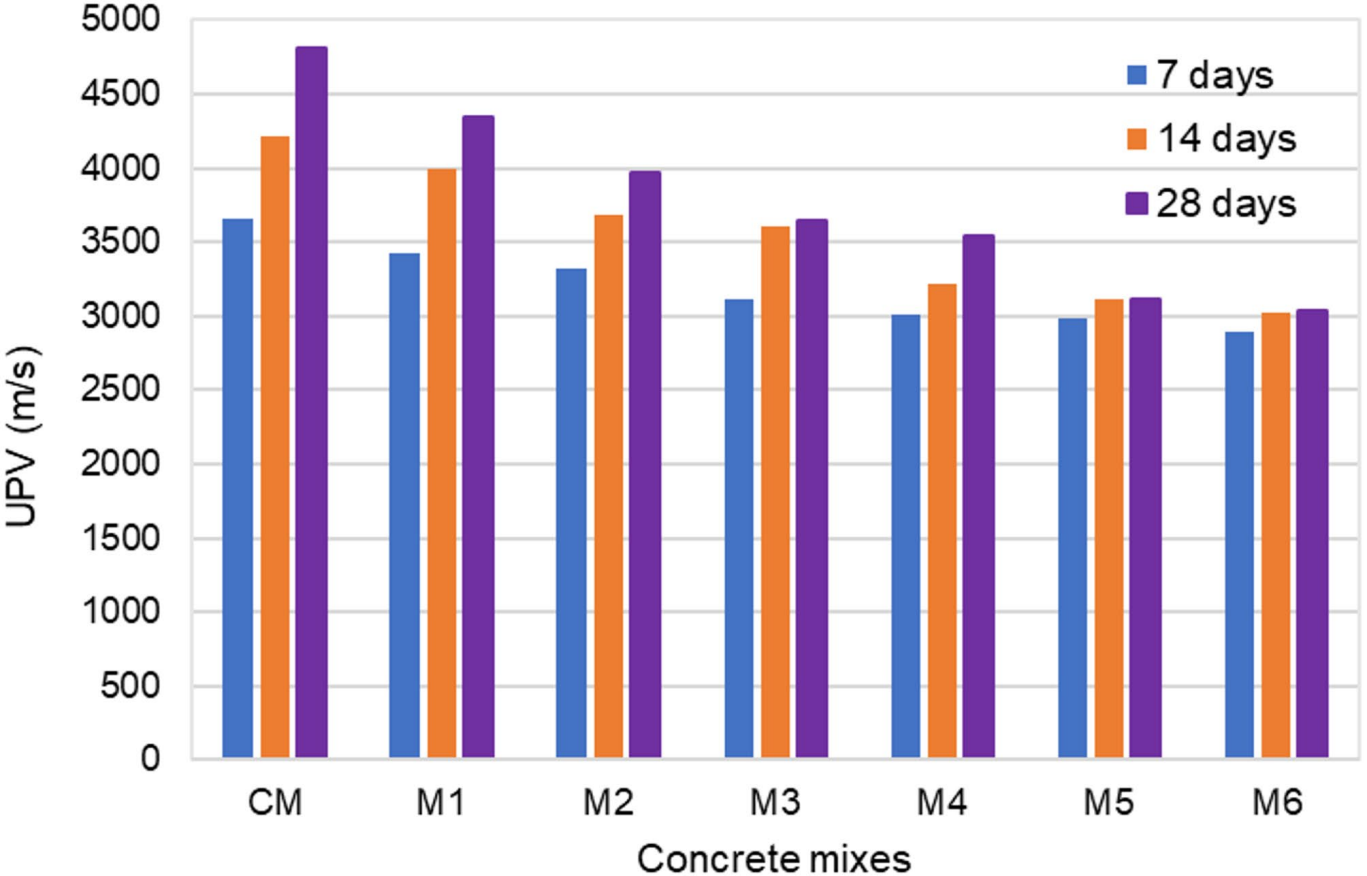
Ultrasonic Pulse Velocity at various curing periods.

Across various curing times, the UPV results show a consistent trend, as illustrated in Fig. 10. The UPV values of every specimen increased with more extended curing periods, which is consistent with the behavior of compressive strength. The UPV increased by 28.9% for the control mix concrete from 7 to 28 days. Likewise, for polyurethane foam concrete with coarse aggregate replacement ranging from 10 to 60%, the UPV increase ranged from 4.45 to 26.7%. This variation is due to the different replacement levels of coarse aggregates with polyurethane foam. Lower replacement percentages show higher UPV values than higher replacement levels, indicating better structural integrity. This is likely because higher replacement levels introduce more polyurethane foam into the matrix, reducing the concrete density and stiffness. As the hydration process continued over an extended curing period, the pores in the concrete mix were gradually filled with hydration products, such as calcium silicate hydrate (C–S–H), resulting in denser and more compact concrete specimens. Consequently, ultrasonic pulses traveled faster through the solid concrete matrix, requiring less time. These findings were in agreement with those reported in earlier research.

### Correlation between UPV and compressive strength

**Fig. 11:**
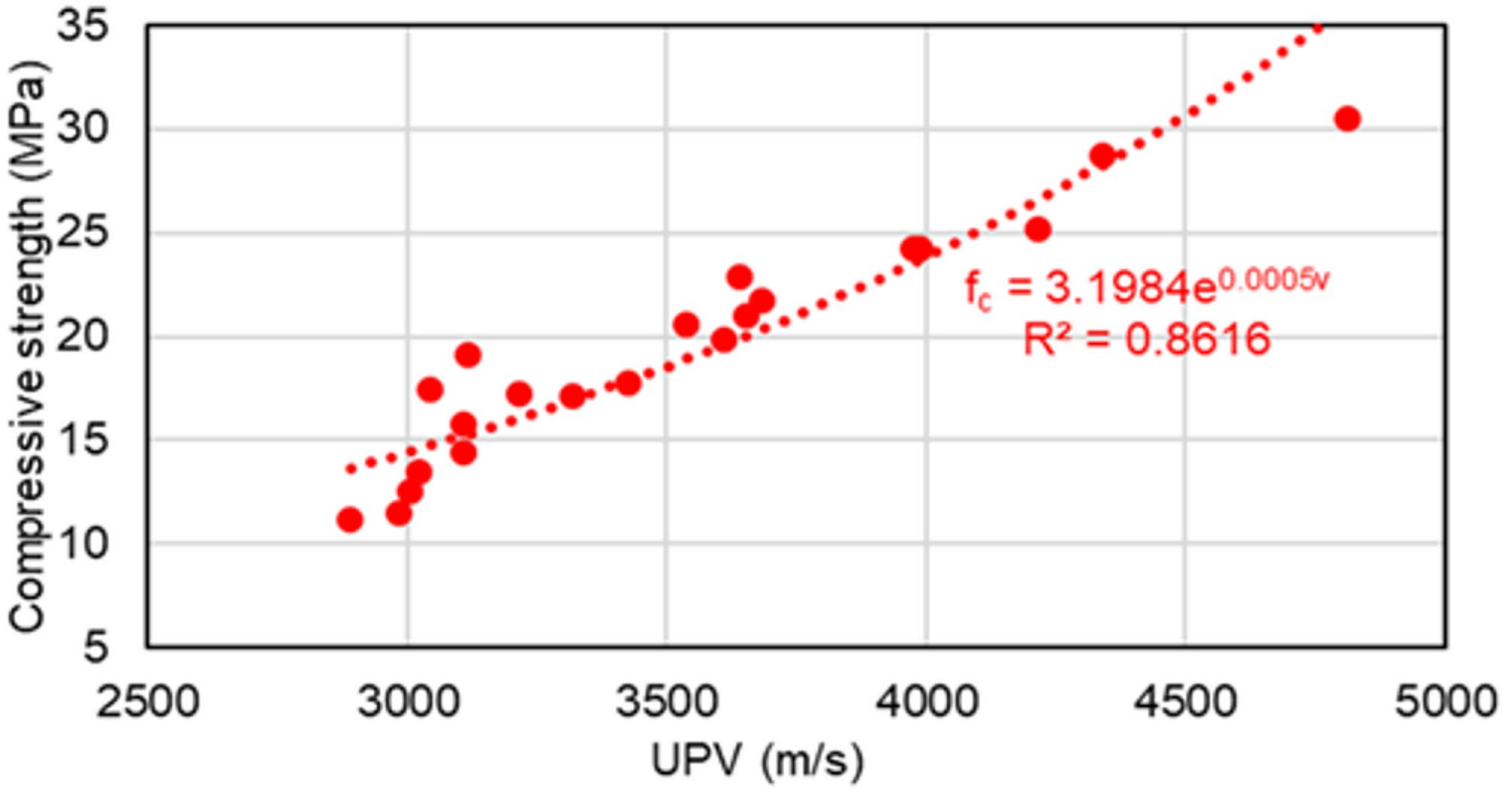
The overall relationship between UPV and compressive strength.

The test findings show that the compressive strength and UPV of polyurethane foam concrete are considerably impacted by the amount of polyurethane foam and the curing periods. Concrete curing periods of 7, 14, and 28 days were used for the experimental investigation. As shown in Fig. 11, all data points were merged and plotted in order to determine the relationship. The plotted test data results demonstrate that the compressive strength of polyurethane foam concrete exhibits an exponential correlation with UPV.

1 $$\:{{f}_{c}=ae}^{bv}$$

where $\:{f}_{c}$ is the compressive strength of concrete (MPa), $\:a$ and $\:b$ are constants derived from experimental data, $\:e$ is the exponential constant, and $\:v$ is the ultrasonic pulse velocity (m/s). Compressive strength was predicted using general empirical formulae based on UPV values:

2 $$\:{{f}_{c}=3.1984e}^{0.0005v}$$

**Fig. 12:**
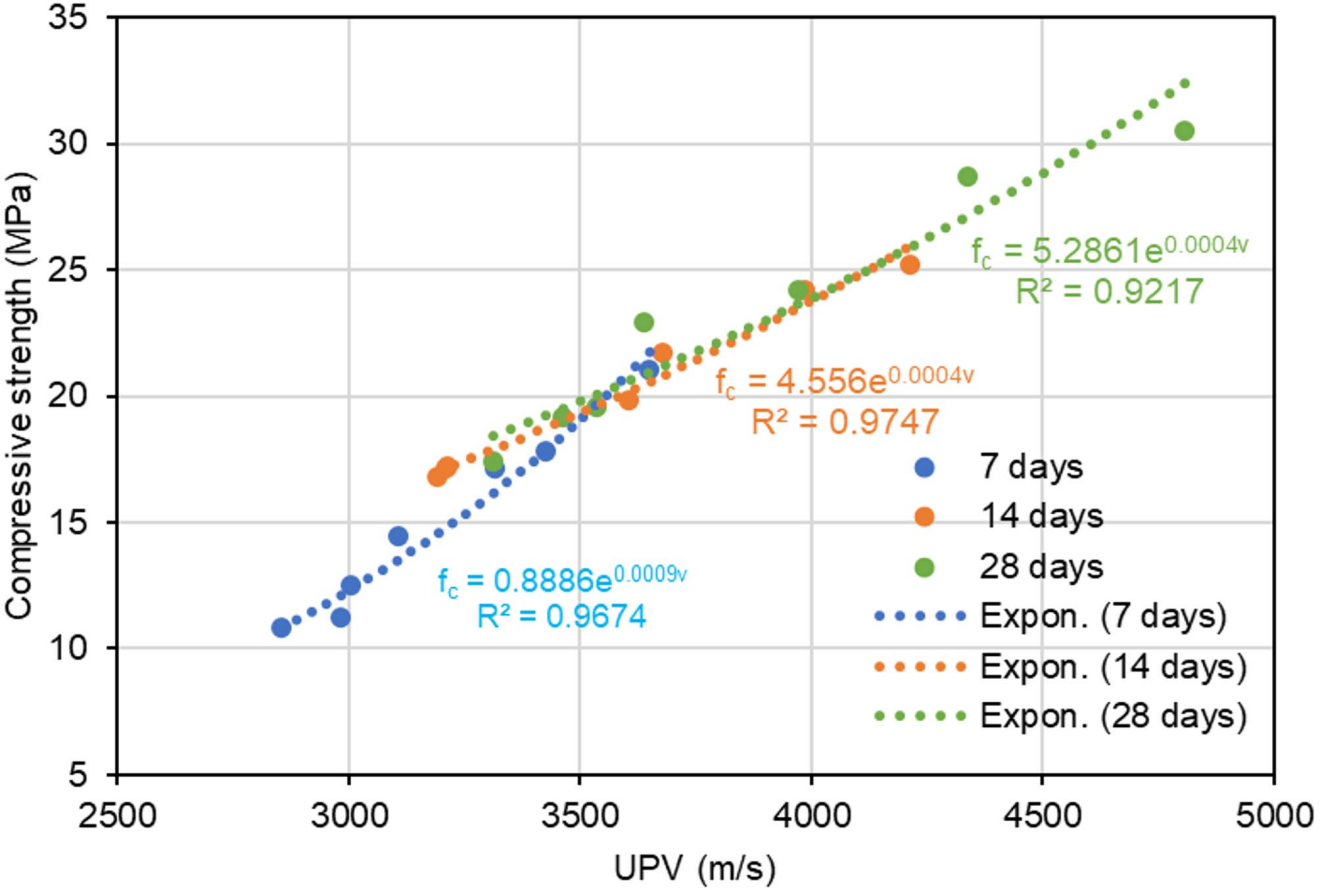
Relationship between compressive strength and UPV in polyurethane foam concrete across different curing periods.

**Fig. 13:**
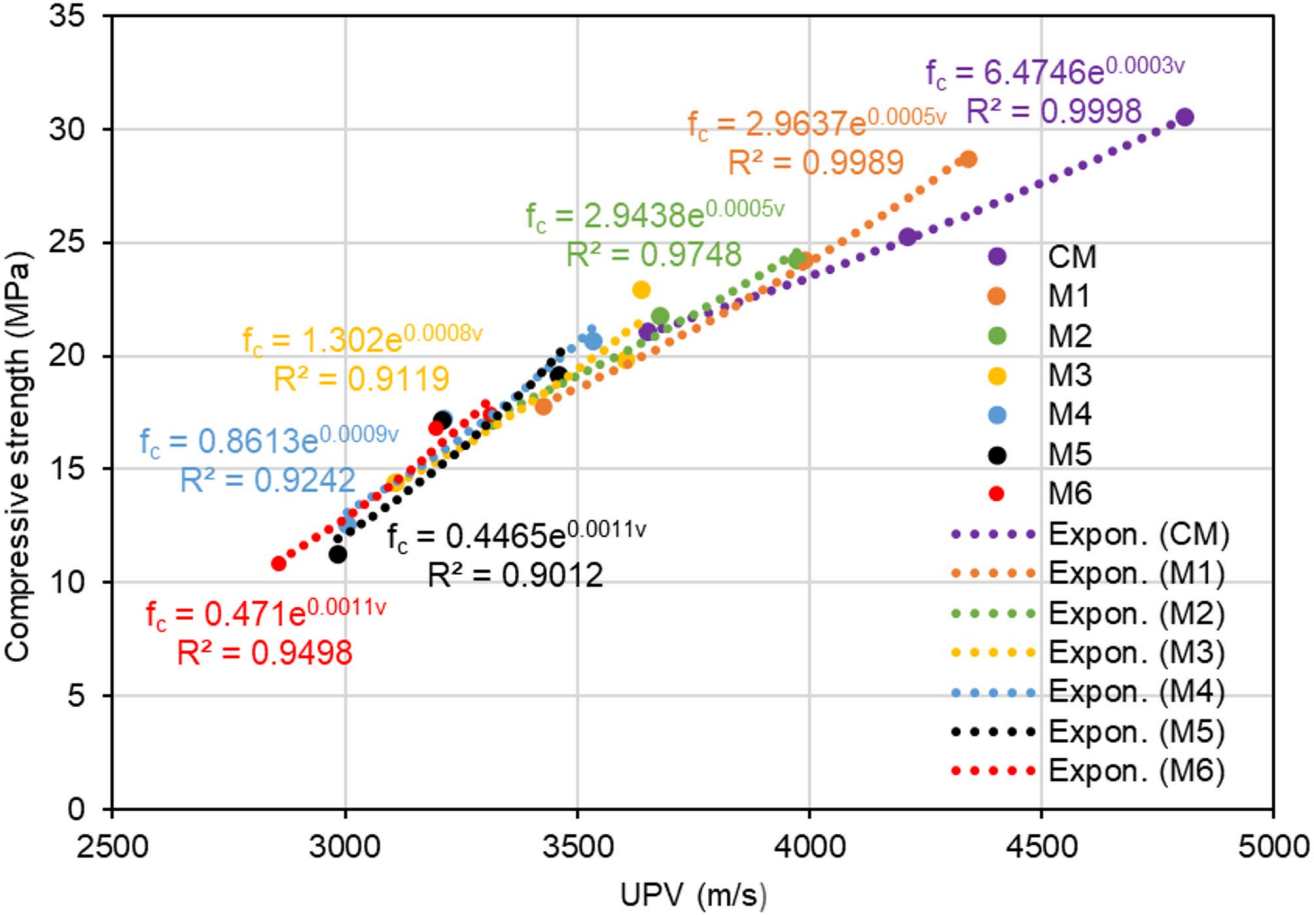
Relationship between compressive strength and UPV in polyurethane foam concrete across different mixes.

The coefficient of determination (R²) for the polyurethane foam concrete mix proportions was around 0.87, demonstrating a robust exponential correlation between compressive strength and UPV, as illustrated in Fig. 11. Furthermore, Fig. 12 illustrates the coefficient of determination (R²) varies between 0.9217 and 0.9747 depending on the curing periods. Figure 13 shows that R² values fluctuate between 0.9012 and 0.9998, depending on the level of polyurethane foam replacement, further demonstrating the variability in compressive strength and UPV based on these factors. In conventional concrete, UPV and compressive strength exhibit a strong positive correlation because denser, stronger concrete allows ultrasonic waves to propagate more quickly. However, in polyurethane foam concrete, this relationship is more complex due to its highly porous and lightweight nature, which significantly influences both UPV and compressive strength. The presence of air voids slows down ultrasonic pulse transmission, resulting in lower UPV values. This correlation aligns with previous research that established an relationship between lightweight concrete compressive strength and UPV, including studies by Fatahi et al.^44^ Shariq et al.^43^ Nikbin et al.^48^ Zhang and F. Aslani^49^ and Shafigh et al.^46^. Compared to these studies, the present study on polyurethane foam concrete demonstrates a better correlation between UPV and compressive strength. This improvement is likely due to the higher compressibility of polyurethane foam aggregates and their enhanced bonding with the cementitious matrix, which significantly improves the transmission of ultrasonic waves.

The empirical equations derived for various curing periods to predict compressive strength based on UPV were provided by Eq. (3), Eq. (4), and Eq. (5).

3 $$\:{{f}_{c}=0.8886e}^{0.0009v}$$

4 $$\:{{f}_{c}=4.5560e}^{0.0004v}$$

5 $$\:{{f}_{c}=5.2861e}^{0.0004v}$$

**Fig. 14:**
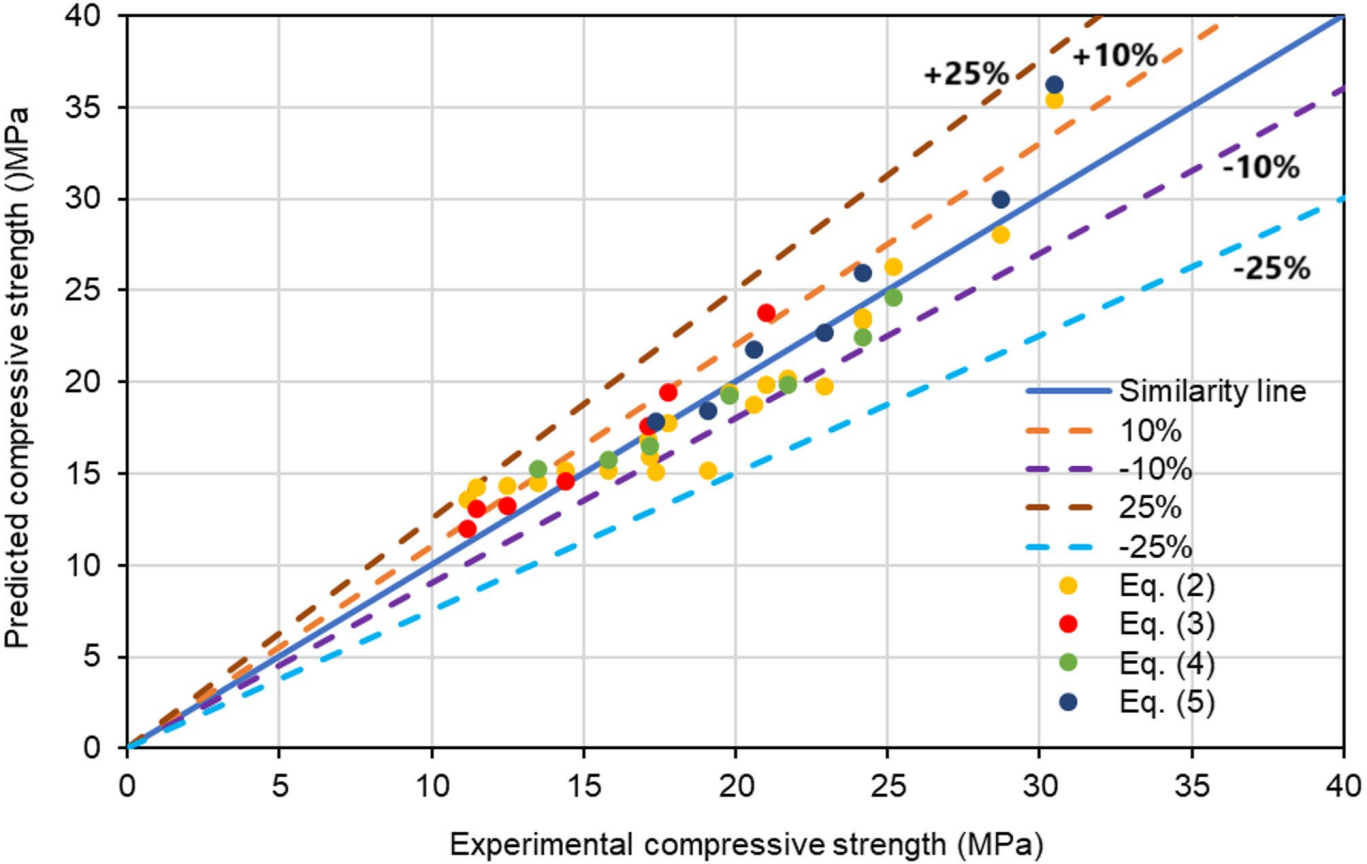
Comparison between the experimental and predicted compressive strength.

The feasibility of polyurethane foam concrete was evaluated by correlating the experimentally obtained compressive strength with the predicted values derived from Eq. (1). Figure 14 compares the experimentally determined compressive strength with the predicted values for polyurethane foam concrete. Most of the data points show a high degree of consistency between the experimental and predicted values, closely matching the 45-degree similarity line. However, a few data points fall just short of the similarity line while still falling within the allowable deviation range of ± 10%. Furthermore, the predicted compressive strengths at 7, 14, and 28 days, calculated using Eq. (3) – Eq. (5), are closely aligned with the similarity line, demonstrating strong agreement with the experimental results. This indicated that the compressive strength of polyurethane foam concrete can be accurately predicted using the proposed exponential equations.

## Conclusions

This study evaluated the suitability of the non-destructive UPV technique for measuring the strength properties of polyurethane foam concrete. Concrete samples were made with different waste polyurethane foam contents (10–60%) and examined at various curing ages (7, 14, and 28 days). The following conclusions are drawn from the findings.

- Replacing natural coarse aggregate with waste polyurethane foam reduced the workability and density of the concrete due to its lightweight and porous nature. Its high porosity led to increased water absorption, which further negatively impacted workability.
- SEM images show improved bonding between the polyurethane foam aggregate and the cementitious matrix, as the cement paste penetrates the surface porosity and rough texture of the polyurethane foam.
- The impact of adding polyurethane foam on UPV is comparable to that on compressive strength; both decrease as the replacement level (10–60%) increases.
- The concrete compressive strength and UPV increase with increasing curing age (7–28 days), however the rate and degree of this growth vary depending on the properties of the concrete.
- An exponential correlation was observed between the UPV and the compressive strength of polyurethane foam concrete. When UPV was plotted against compressive strength data for various curing periods and mixes, exponential relationships with strong correlation coefficients (0.9012 < R² < 0.9998) were observed.
- A high degree of agreement between the experimental and predicted test results confirmed that the suggested empirical equations successfully evaluated the compressive strength of polyurethane foam concrete samples.

According to the current study, the UPV technique can be used to quickly evaluate the properties of polyurethane foam concrete on-site and in the lab. Heavy load-bearing structures, such as foundations and bridges, require high strength, which limits the use of polyurethane foam concrete in these applications. While most test results met the standards for lightweight concrete construction, further research is needed to enhance its mechanical performance through the use of physical and chemical admixtures. Future studies should explore the impact of controlling parameters, including particle size distribution, curing regimes, and the incorporation of physical, mineral, and fiber additives, to optimize the properties, UPV- strength relationship and broaden the applicability of polyurethane foam concrete.

## Data Availability

The datasets used and/or analysed during the current study available from the corresponding author on reasonable request.

## Abbreviations

UPV: Ultrasonic Pulse Velocity
IS: Indian Standard
SEM: Scanning Electron Microscopy
CM: Control Mix
EDAX: Energy Dispersive X-ray Analysis
ACI: American Concrete Institute
EN: European Standards
ITZ: Interfacial Transition Zone
Eq: Equation
C: Carbon
O: Oxygen
Ca: Calcium
Si: Silicon
Al: Aluminum
Fe: Iron
K: Potassium
